# Physiological consequences of consuming low-energy foods: herbivory coincides with a stress response in Yellowstone bears.

**DOI:** 10.1093/conphys/coab029

**Published:** 2021-07-30

**Authors:** David Christianson, Tyler H Coleman, Quint Doan, Mark A Haroldson

**Affiliations:** 1 Department of Ecosystem Science and Management, University of Wyoming, Laramie, WY 82071, USA; 2 Sequoia-Kings Canyon National Park, National Park Service, 47050 Generals Highway, Three Rivers, CA 93271, USA; 3 School of Forestry and Environmental Studies, Yale University, 370 Prospect Street, New Haven CT 06511, USA; 4 U.S. Geological Survey, Northern Rocky Mountain Science Center, Interagency Grizzly Bear Study Team, 2327 University Way, Suite 2, Bozeman, MT 59717, USA

## Abstract

Meat, fruit, seeds and other high-energy bear foods are often highly localized and briefly available and understanding which factors influence bear consumption of these foods is a common focus of bear conservation and ecology. However, the most common bear foods, graminoids and forbs, are more widespread but of lower quality. We poorly understand how herbage consumption impacts bear physiology, such as endocrine system function that regulates homeostasis and stress responses. Here, we described bear diets with a novel approach, measuring the concentration of chlorophyll in bear scats (faecal chlorophyll) to index the proportion of the recent diet that was composed of leaves from graminoids and forbs. We measured faecal chlorophyll and faecal cortisol in 351 grizzly (*Ursus arctos*, *n* = 255) and black bear (*Ursus americanus*, *n* = 96) scats from Yellowstone National Park in 2008–2009. We compared models of faecal chlorophyll and faecal cortisol concentrations considering the effects of spatial, dietary, scat and bear-specific factors including species. Faecal chlorophyll levels were the strongest predictor of faecal cortisol in a manner that suggested an endocrine response to a low-energy diet. Both compounds were highest during the spring and early summer months, overlapping the breeding season when higher energy foods were less available. Effects of scat composition, scat weathering, bear age, bear sex, species and other factors that have previously been shown to influence faecal cortisol in bears were not important unless faecal chlorophyll was excluded from models. The top models of faecal chlorophyll suggested grazing was primarily influenced by spatial attributes, with greater grazing closer to recreational trails, implying that elevated cortisol with grazing could be a response to anthropogenic activity. Our results confirm that higher stress hormone concentrations correspond with lower quality diets in bears, particularly grazing, and that faecal chlorophyll shows promise as a metric for studying grazing behaviour and its consequences.

## Teaser

Stress hormone concentrations in bear scats are best explained by chlorophyll in the scat, which describes the amount of herbaceous vegetation in the diet. Bears that grazed more on grasses and forbs had the highest stress hormone concentrations. Bears grazed more near trails.

## Introduction

To survive, grow and reproduce, most species appear able to use cues from their environment to adaptively respond to current conditions. In vertebrates, many cues influence neuroendocrine activity in the hypothalamic–pituitary–adrenal (HPA) axis triggering several physiological responses. Stress responses initiated by the HPA are those triggered by the release of cortisol and other glucocorticoids from the adrenal cortex (Sapolsky *et al.*, 2000). HPA regulation of glucocorticoid release can influence behaviour and life history tactics to adaptively meet the demands posed by external stressors (Boonstra *et al.*, 2001; Love and Williams, 2008).

Stressors and their demographic consequences can be identified by describing HPA activity in free-ranging animals, facilitating our understanding of the evolution, ecology or conservation challenges of many species (Boonstra, 2002; Sih *et al.*, 2004; Busch and Hayward, 2009; Creel *et al.*, 2013). One commonly observed effect of cortisol is increased gluconeogenesis: catabolization of endogenous energy stores to support muscle and other tissues affecting energy balance (Boonstra *et al.*, 1998). Increased gluconeogenesis could facilitate immediate behavioural responses to an acute short-term stressor such as fleeing from an attacking predator (Wingfield, 2003). Over longer time scales, cortisol levels appear to play an important role in long-term energy balance, an effect that is most conspicuous in species that endure periods of hyperphagia and hibernation (Romero *et al.*, 2008). Owing to the multi-functional role of cortisol, it is difficult to interpret the significance of observed cortisol levels as a response to a stressor in free-ranging animals, particularly without a nutritional context.

Bears (*Ursus spp*) annually undergo considerable change in body mass due to hyperphagia in late summer and fall to replace body stores lost during winter hibernation and periods of spring and early summer undernutrition (Schwartz *et al.*, 2014). Bears can meet or exceed nutritional demands by being flexible foragers—regularly consuming mammals, fish, invertebrates, herbaceous matter, fruits, seeds and fungus (Gunther *et al.*, 2014). Studies of bear foraging and its physiological consequences have often focused on the most energy-dense foods, such as animal meat, fruit or pine nuts (Bryan *et al.*, 2013; Costello *et al.*, 2014; Teisberg *et al.*, 2014), which are often only briefly available and localized, requiring bears to temporarily congregate on concentrated food sources and traverse long distances between them (Fortin *et al.*, 2013; Costello *et al.*, 2014). The need to move widely among concentrated food resources that must be shared with other bears creates ample opportunities for exposure to social and external stressors, including encounters with humans along roads or trails that must be crossed or travelled by bears. While consumption of high-energy foods is probably critical for bear survival, it can be difficult to parse their physiological significance due to their brief and sometimes unpredictable availability and confounding effects of close encounters with humans and other bears during consumption (Ben-David *et al.*, 2004).

Grazing herbaceous vegetation is a ubiquitous foraging behaviour in Yellowstone bears but its physiological consequences are even less well understood. Gunther *et al*. (2014) showed that of 39 food items described in 11 478 Yellowstone grizzly bear (*Ursus arctos*) scats examined from 1943 to 2009, graminoids occurred in 58.4% with many other herbaceous plants also occurring frequently. Grass alone occurred nearly four times more frequently than the second-most common food item—ants (Gunther *et al.*, 2014). Additionally, graminoids and forbs are overwhelmingly more abundant than other bear foods and are the most common foods selected at bear foraging sites (Fortin *et al.*, 2013). However, graminoids and forbs generally have much lower available fat, carbohydrate and protein dry matter concentrations of other bear foods. Availability of these nutrients is lower in grasses than in other foods due to high water and fibre content, lowering digestibility (Pritchard and Robbins, 1990; Craighead *et al.*, 1995). For example, the gross energy content of whole ground squirrels (*Spermophilius columbianus*) and white clover (*Trifolium repens*) on a dry matter basis is similar—22.11 kilojoules/g and 20.22 kilojoules/g, respectively). However, a meal composed of 5 kgs of ground squirrels contains 24 606 kilojoules of digestible energy, while a meal composed of 5 kgs of white clover contains only 7270 kilojoules of digestible energy. The disparity arises due to differences in water content and digestibility (Pritchard and Robbins, 1990). Diets comprised mostly of herbaceous vegetation are less likely to provide sufficient energy to prevent hypoglycemia and minimize gluconeogenesis, the mobilization of stored fat as blood sugar for catabolism, and the effects of cortisol on these processes may play an import role in any response (Reeder and Kramer, 2005).

While grazing is perhaps the most common foraging strategy used by bears and the extent of grazing likely strongly influences nutrient balance, the relationship between grazing and bear physiology remains poorly understood. To test the hypothesis that diet quality itself may influence HPA axis activity, we measured grazing activity and cortisol levels in grizzly and black bear scats to assess endocrine responses associated with consuming herbaceous diets. We also considered that grazing may have affected exposure to acute stressors, such as interactions with conspecifics or humans on roads and trails.

## Materials and Methods

We studied diet selection and stress hormone levels in grizzly bears and black bears in Yellowstone National Park, WY, USA. The bears and scats involved in this study were originally included in research detailing dietary adjustability and human interactions (Coleman *et al.*, 2013; Fortin *et al.*, 2013). Trapping and GPS collaring of bears occurred from April to November from 2006 to 2009 by the Interagency Grizzly Bear Study Team using methods described previously (Coleman *et al.*, 2013). Since 1997, bear capture and handling procedures were reviewed and approved by the Animal Care and Use Committee of the United States Geological Survey; procedures conformed to the Animal Welfare Acts and to United States Government principles for use and care of vertebrate animals in testing, research and training. Grizzly bear captures were conducted under United States Fish and Wildlife Service Endangered Species Permit [Section (i) C and D of the grizzly bear 4(d) rule, 50 CFR17.40 (b)], Yellowstone National Park Research Permit YELL-00073 and Grand Teton National Park Research Permit GRTE 1990-Sci-0003. Weekly telemetry flights occurred during the study period to collect data stored in the collars.

In 2008 and 2009, from 15 April to 16 November, we collected bear scat by randomly selecting a bear and a date from the previous week’s GPS collar data. Field crews searched each GPS collar point visited by the bear on the selected date but only for fixes separated by at least 1 hour (some GPS collars collected more than one fix per hour). Field crews delayed searching a site for faecal samples if the bear still occupied the location. Scat samples were collected within a 20-m radius of the GPS fix and were assumed to belong to the collared bear. When searching the prior locations of adult females that had been confirmed to be with cubs, we did not sample from faecal piles that were small in volume or diameter to minimize sampling from cubs. No samples were collected more than 14 days after a GPS fix, and we recorded the time lag between GPS fix and faecal collection to account for any effects of weathering on scats in our models. Field teams froze the scat samples at −20°C until laboratory analysis. We assumed faecal samples collected within 20 m on the same day belonged to the same bear or female-cub group. For each faecal sample, we noted bear identity, bear age, bear sex, bear species, scat age (the time lag between a GPS collar fix and the faecal collection), Julian day of GPS fix, time of day of GPS fix, elevation, latitude and longitude.

**Figure 1:**
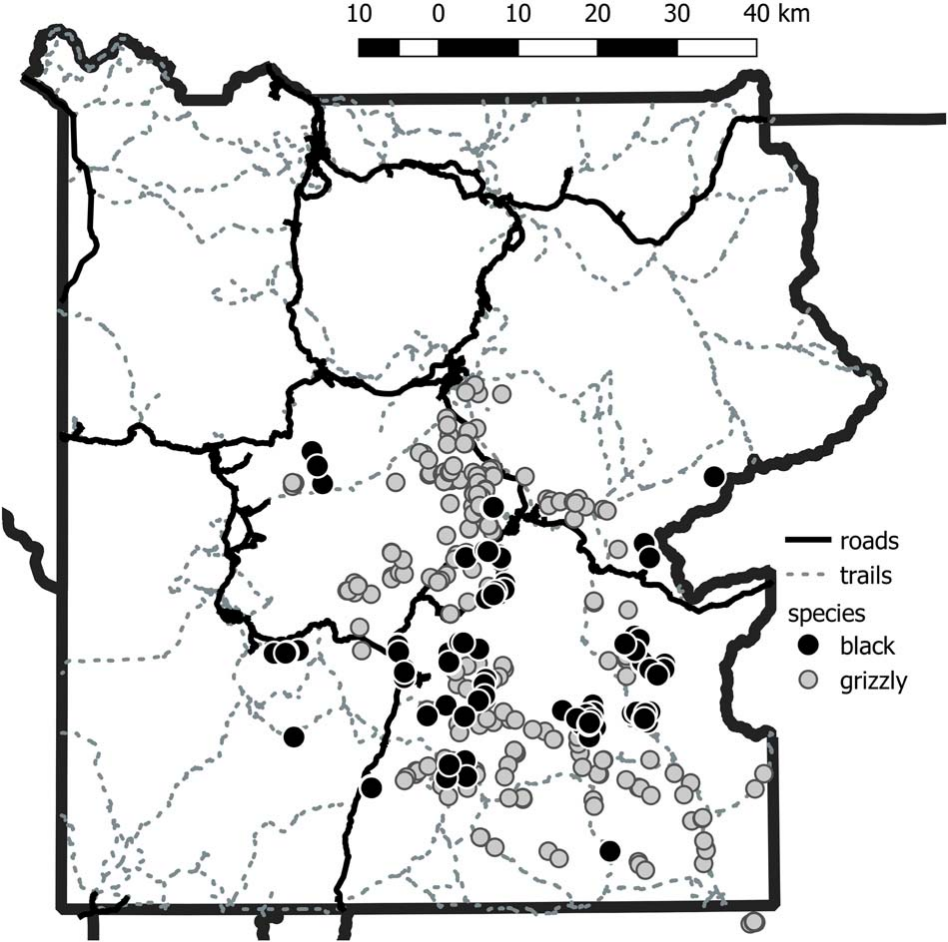
Location of trails and roads in Yellowstone National Park, Wyoming, Montana and Idaho, and grizzly bear and black bear scat samples collected from 2008 to 2009. A random sample of 10 000 points (not shown) was used to estimate median distances to roads and trails across the entire park.

Using a geographic information system (R Core Team, 2020), we extracted the distance from the nearest road and recreational trail to each faecal sample, as well as the land cover (U.S. Geological Survey, 2011) and the Normalized Difference Vegetation Index (NDVI) at the faecal collection site (Fig. 1). The NDVI is an index of the abundance of photosynthetically active vegetation and can describe the availability of green forbs and grasses for herbivory (Pettorelli *et al.*, 2005). However, the ability of satellite-based NDVI data to index green biomass in the herb layer is hindered by forest over-story. When the USGS GAP Land Cover Level 3 Ecological System at a faecal collection site was identified as turf, grassland, shrubland, meadow, sagebrush-steppe or recently disturbed forest, we extracted the NDVI value at that site. When the USGS GAP Land Cover Level 3 Ecological System at a faecal collection site was forested or developed, we used the mean NDVI measure at all non-forested and undeveloped faecal collection sites. We used a smoothed, gap-filled NDVI product derived from the MODIS instrument on both the Aqua and Terra satellites, which provided a measure of green biomass availability at 250-m resolution approximately every 8 days (Spruce *et al*. 2016).

For each scat location, we also extracted an index of annual grizzly bear density resolved to 14-km by 14-km grid squares across the study area (Bjornlie *et al.*, 2014). The grizzly bear density index was derived from radio telemetry of collared bears from 1983 to 2012 to estimate the number of home ranges that completely overlapped a grid cell. Finally, we categorized landcover classifications at scat collections as grassland, shrubland or forested to examine the effects of vegetative cover.

### Faecal analysis

We categorized each faecal sample according to the most macroscopically conspicuous food item in the scat: meat, seed *(Pinus albicaulis* cone seeds), vegetation or mixed (mixed items, fruit, insects, unknown); however, this categorization did not necessarily reflect the most common food item in the sample (see Results). We used scat type to describe the diet as well as properties of the scat because high-fibre, plant-based diets are generally retained in the gut for much shorter lengths of time than meat, grain or fruit-based diets in bears (Pritchard and Robbins, 1990; Elfström *et al.*, 2013). Differences in gut retention times could affect the degree of exposure to circulating glucocorticoids and the temporal lag between events that trigger a stress response and defecation (Wheeler *et al.*, 2013). We boiled a known mass (~0.2 g) of homogenized feces in 95% ethanol to extract steroid hormones and chlorophyll before reconstituting extracts in 1 ml of 100% methanol, following published methods (Creel *et al.*, 2002; Christianson and Creel, 2009).

The chlorophyll concentration of common forage grasses and forbs from the Greater Yellowstone Ecosystem has been shown to strongly correlate with digestibility, energy content and nitrogen content of the forage (Christianson and Creel, 2014). Faecal chlorophyll in herbivores corresponds strongly with seasonal cycles in primary productivity (Lowry and Schlink, 1995; Lane and Hassall, 1996; Szymczak-Zyla *et al.*, 2006; Christianson and Creel, 2009) and other common faecal indices of diet quality like faecal nitrogen (Christianson and Creel, 2014). To quantify faecal chlorophyll, we measured the optical density of faecal extracts at 666 nm (OD_666_) – the wavelength at which chlorophyl *a* is most active when diluted in methanol (Lichtenthaler and Wellburn, 1983). We then corrected for turbidity (OD_666_ – OD_750_) and adjusted for dilution of the extract and the dry weight of the extracted sample so that faecal chlorophyll is expressed as the optical density at 666 nm per gram of dry feces or OD per g (Deijs and Bosman, 1955; Szymczak-Zyla *et al.*, 2006).

We measured faecal cortisol concentrations in faecal extracts using an enzyme-linked immunoassay kit (Enzo Life Sciences ADI-900-071) with a cortisol antibody with broad cross reactivity to cortisol (100%), prednisolone (122.35%), corticosterone (27.68%), 11-deoxycortisol (4.0%), progesterone (3.64%), prednisone (0.85%), testosterone (0.12%) androstenedione, cortisone and estradiol(each <0.10%) and that has been validated in feces for several marsupial and eutherian carnivores, herbivores and omnivores (Rothschild *et al*. 2010; Ezenwa *et al*. 2012; Forristal *et al*. 2012; Creel *et al*. 2013; Schwanz and Robert 2014; Running 2015; Cinque *et al*. 2017; Lemos *et al*. 2020). We initially assayed all extracts at 1:8 dilution. We re-assayed each extract at higher or lower dilutions to improve precision if binding was not near the middle of the standard curve, using dilutions spanning 1:3 to 1:20. The ELISA demonstrated high sensitivity (56.72 pg/ml) relative to the working concentrations of extracts (4835 pg/ml). The intra-assay coefficient of variation was 5.9% and the inter-assay coefficient of variation was 6.1%.

Weathering is known to influence faecal cortisol concentrations, possibly due to microbial metabolism of cortisol in the feces after defecation (Millspaugh and Washburn, 2004; Stetz *et al.*, 2013; Dalerum *et al.*, 2020). To measure the effects of weathering, we opportunistically collected 51 fresh faecal samples from road-side black bears and grizzly bears. From 23 May until 20 September 2007, we weathered these 51 faecal samples, *in situ*, and subsampled (if enough sample remained) after 6, 24, 48, 72, 96, 120, 144, 168 and 192 hours of weathering. Linear trends in (log) faecal cortisol within samples showed a weak increase with weathering (weighted mean slope ± SE in log faecal cortisol regressed on hours of weathering time: 0.0015 ± 0.0003) equivalent to an increase of 0.84 ng/g faecal cortisol per 24 hours of weathering, or a 3.5% change from mean faecal cortisol. Faecal chlorophyll weakly decreased with weathering (mean slope ± SE: −0.00043 ± 0.00044), equivalent to a 3.5% decline from mean faecal chlorophyll per 24 hours of weathering. Most (67%) samples were collected within 7 days of defecation but we considered scat age, i.e. days between GPS fix and faecal collection, in our modelling to account for weathering effects. We also included scat water because precipitation on scats has been shown to decrease faecal cortisol concentrations.

### Data analysis

We compared generalized linear models within and across six categories of candidate models of faecal cortisol that included factors identified in the literature as affecting bear diets, stress responses or behaviour (Bryan *et al.*, 2013; Coleman *et al.*, 2013; Stetz *et al.*, 2013; Costello *et al.*, 2014; Schwartz *et al.*, 2014). Our candidate model set was composed of spatial models (bear density, elevation, distance to roads and distance to trails), bear-specific models (species, sex and age class), diet models (faecal chlorophyll and scat type) and null models (Julian day, time of day, scat age, scat water and scat type) that, collectively, allowed us to examine how potential acute stressors, such as bear density or road proximity, interacted with diet quality to determine cortisol levels. Within each set of candidate models (except global models), we fit each possible explanatory variable by itself and also alongside all other explanatory variables within the set. We fit all candidate models with scat attributes to account for variation in faecal cortisol that may have been due to differences in scat chemistry or digestive kinetics (Evans *et al.*, 2014). Consequently, we also considered models with only scat attributes in our candidate set of null models. Because of seasonal patterns in temperature and faecal cortisol, and because circulating cortisol levels vary over 24-hour and seasonal cycles (Ware *et al.*, 2013), we included linear and quadratic effects of Julian day and the effects of time of defecation (time of GPS fix) in all candidate models and in a null model with only Julian day and time effects. Finally, we also included an intercept-only null model and two global models with all variables (i.e. one global model with faecal chlorophyll but without scat type and one global model with scat type but without faecal chlorophyll, which were highly collinear as descriptors of diet composition). We fit all models as generalized mixed-effects models with a random effect for collection group (with all scats collected within 3 days of each other at a single bear’s consecutive relocations were grouped into the same collection group). We log transformed faecal cortisol and faecal chlorophyll. We square-root transformed distance from the nearest road and recreational trail. We cosine transformed time of day. We standardized distance from the nearest road, distance from the nearest recreational trail, scat age, elevation and Julian day before fitting models, i.e. we transformed these variables to have a mean of 0 and standard deviation of 1. We normalized NDVI to range from 0 to 1.

**Figure 2:**
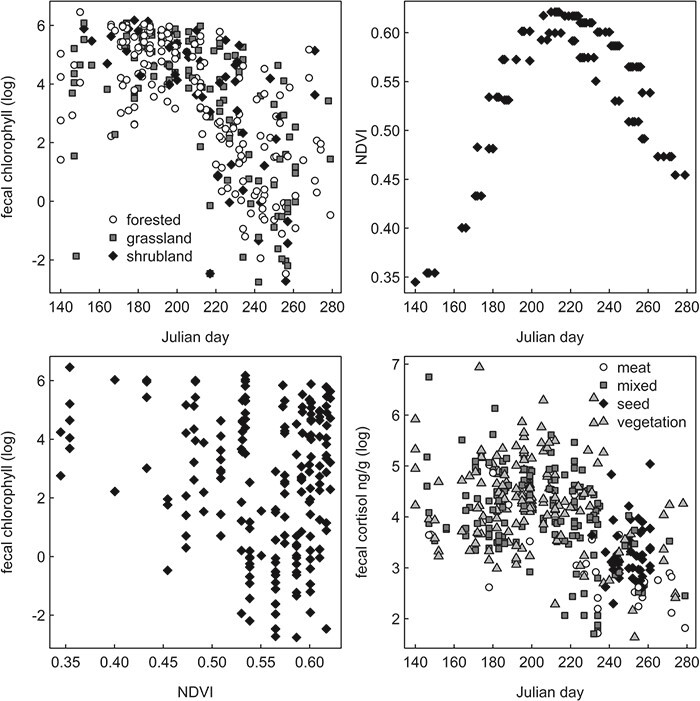
Seasonal trend in faecal chlorophyll by vegetation cover at faecal collection sites (upper left), seasonal trend in the NDVI at grassland and shrubland faecal collection sites only (upper right), relationship between faecal chlorophyll and NDVI at grassland and shrubland sites only (lower left) and seasonal trend in faecal cortisol by primary scat component for grizzly bear and black bear scats collected in Yellowstone National Park from 2008 to 2009.

We compared generalized linear mixed-effects models of faecal chlorophyll, using a similar candidate model set as for faecal cortisol with the following differences. We did not include scat type in any of our candidate models of faecal chlorophyll because both scat type and faecal chlorophyll were highly collinear descriptors of diet composition. We included one candidate spatial model of faecal chlorophyll with NDVI as an explanatory variable to test whether plant phenology described dietary chlorophyll, as it does in large herbivores (Christianson and Creel, 2009). We also included NDVI in a global model of faecal chlorophyll. We did not include time of day in any faecal chlorophyll models because we assumed scats represented a mixture of many foraging choices collected throughout the previous 24 hours. We fit all models by maximum likelihood using the *lmer* packge in R. We used Akaike’s Information Criterion corrected for small sample size (AICc) to select the most parsimonious model to identity whether spatial, bear-specific, dietary or scat-specific attributes best explained variation in faecal cortisol and faecal chlorophyll.

## Results

**Figure 3:**
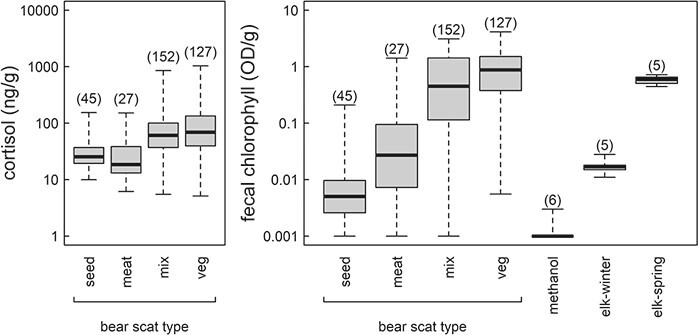
Cortisol (left panel) and chlorophyll (right panel) concentration in faecal extracts from black bears and grizzly bears in Yellowstone National Park with range (whiskers), interquartile range (box) and median (bar) shown. Chlorophyll *a* absorbs light most intensely at 666 nm and concentration is indexed by the absorption of light at this wavelength. Faecal samples from bears were grouped based on macroscopic determination of the most common item present in the scat but most faecal samples contained multiple items. The optical density of the diluent used in all assays, methanol and faecal extracts from a strict herbivore, Yellowstone elk (*Cervus elaphus canadensis*) in winter and spring are shown for reference. Numerals above each whisker are sample sizes.

Faecal chlorophyll and faecal cortisol from 351 bear scats (96 male black bear, 136 female grizzly bear and 145 male grizzly bear) indicated high variation and seasonal trends (Fig. 2). The highest chlorophyll levels occurred during the spring–summer period, which generally correlated with the increasing NDVI and was also associated with the breeding season for both species of bears. During the later post-breeding hyperphagic period as NDVI declined, chlorophyll levels were highly variable but generally declining. Interestingly, male grizzly bears had higher faecal chlorophyll (log OD per g) than male black bears (4.06 and 3.22, respectively; Welch’s t_204._ = −3.01; *P* = 0.002) and female grizzly bears (4.06 and 2.89, respectively; Welch’s t_236_ = −4.10; *P* < 0.001): faecal cortisol exhibited a similar seasonal trend as faecal chlorophyll. Variation in faecal cortisol spanned several orders of magnitude with faecal samples that were primarily vegetation showing the highest faecal cortisol levels (Fig. 3). Male grizzly bears had higher faecal cortisol (log ng per g) than female grizzly bears (4.12 and 3.77, respectively; Welch’s t_240_ = −3.05; *P* = 0.002) but not male black bears (4.12 and 4.00, respectively; Welch’s t_205_ = −1.10; *P* = 0.273). The top model of faecal cortisol included a strong positive effect of faecal chlorophyll (coefficient estimate ± SE: 0.198 ± 0.023, *t* = 8.58) along with no effect of scat age (0.001 ± 0.043, *t* = 0.03), positive linear (1.19 ± 0.54, *t* = 2.20) and negative quadratic (−1.45 ± 0.55, *t* = −2.64) effects of Julian day, no effect of time of day (−0.022 ± 0.051, *t* = −0.44) and a strong negative effect of scat water content (−0.268 ± 0.04, *t* = −6.83). The top model was the only candidate model strongly supported by the data, receiving 1.00 of the Akaike weight. Faecal cortisol models that did not include faecal chlorophyll were at least 53 AICc units worse than the top model (Table 1).

**Table 1:** Model selection results for generalized linear mixed-effects model of faecal cortisol in black bear and grizzly bear faecal samples collected opportunistically from road and trail-side bears and from site searches at GPS collar locations in Yellowstone National Park in 2008–2009

Faecal cortisol model description^a^	Model category^b^	k	AICc	ΔAICc	Weight	Log likelihood
day + day^2^ + time + scat water + scat age + chlorophyll	D	9	726.24	0.00	1.00	−353.85
day + day^2^ + time + scat age + scat water + chlorophyll + age + sex + species + elevation + road + trail + density + cover	G	18	737.37	11.13	0.00	−349.65
day + day^2^ + time + scat age + scat water + scat type	N, D	11	779.24	53.00	0.00	−378.23
day + day^2^ + time + scat age + scat water + trail	S	9	781.76	55.53	0.00	−381.62
day + day^2^ + time + scat age + scat water + road + trail	S	10	782.92	56.68	0.00	−381.13
day + day^2^ + time + scat age + scat water + scat type + age + sex + species + elevation + road + trail + density + cover	G	20	785.14	58.90	0.00	−371.30
day + day^2^ + time + scat age + scat water + density	S	9	788.55	62.31	0.00	−385.01
day + day^2^ + time + scat water	N	7	789.14	62.91	0.00	−387.41
day + day^2^ + time + scat age + scat water + elevation	S	9	791.75	65.52	0.00	−386.61
day + day^2^ + time + scat age + scat water + road	S	9	792.07	65.84	0.00	−386.77
day + day^2^ + time + scat age + scat water + elevation + density + cover	S	12	792.30	66.06	0.00	−383.69
day + day^2^ + time + scat age + scat water + species	B	9	792.51	66.28	0.00	−386.99
day + day^2^ + time + scat age + scat water + sex	B	9	792.81	66.57	0.00	−387.14
day + day^2^ + time + scat age + scat water + age	B	9	792.97	66.73	0.00	−387.22
day + day^2^ + time + scat age + scat water + cover	S	10	793.31	67.07	0.00	−386.33
day + day^2^ + time + scat type	N	9	795.85	69.61	0.00	−388.66
day + day^2^ + time + scat age + scat water + age + sex + species	B	11	796.73	70.49	0.00	−386.98
day + day^2^ + time	N	6	807.04	80.80	0.00	−397.40
day + day^2^ + time + scat age	N	7	807.23	80.99	0.00	−396.451
Intercept only	N	3	873.11	146.87	0.00	−433.519

^a^Model components are Julian day (day), time of day (time), optical density at 666 nm of scat extract (chlorophyll), age of bear (age), sex of bear (sex), species of bear (species), distance to the nearest road (road), distance to the nearest trail (trail), cover (grassland, shrubland and forest), primary dietary component of the sample (scat type), the weathering time between sample collection and defecation (scat age), water content of sample (scat water), the elevation of the sample collection (elevation) and an index of the density of grizzly bears (density).

^b^Candidate models were ordered into the following five categories: global models (G), null models (N), diet models (D), spatial models (S) and bear models (B).

**Figure 4:**
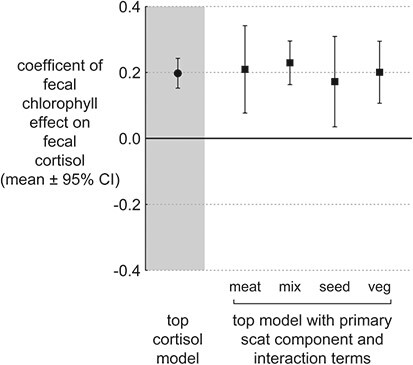
Coefficient estimate for the effects of faecal chlorophyll on faecal cortisol in the top model and the top model with separate main effects of primary scat components and their interaction with faecal chlorophyll. Positive effects of faecal chlorophyll on faecal cortisol were not an artefact of dietary composition.

To further test whether the effects of faecal chlorophyll on faecal cortisol were an artefact of physico-chemical differences in scat types, we refit the top model with separate intercept terms for each primary scat component type (meat, seed, vegetation, other) and separate slope terms for faecal chlorophyll effects on cortisol within each scat component type. We examined whether these intercept and slope coefficients, describing the effect on faecal cortisol of scat type and the scat type interaction with faecal chlorophyll, were consistent across primary scat types. The intercept terms and the terms describing effects of faecal chlorophyll on faecal cortisol were no different from each other (i.e. overlapping 95% CI) nor were they different from the terms in the top model suggesting consistent effects of faecal chlorophyll on cortisol regardless of the macroscopic composition of the faecal sample. (Fig. 4).

Chlorophyll in bear scats was highly variable even within scats grouped by primary diet item, including some samples showing almost no chlorophyll and other samples with chlorophyll levels comparable to that seen in obligate herbivores (Fig. 3). The two top models were spatial models and were within 0.65 AICc units of each other and suggested both roads and trails were important. The second-ranked model that included distance from both roads and trails showed positive linear (coefficient estimate ± SE: 2.96 ± 1.42, *t* = 2.08) and negative quadratic effects of Julian day (−4.00 ± 1.42, *t* = −2.81), weak effects of scat age (0.099 ± 0.111, *t* = 0.892), strong positive effects of faecal water (0.376 ± 0.083, *t* = 4.54), a positive effect of distance from the nearest road (0.154 ± 0.127, *t* = 1.21) and a strong negative effect of distance from the nearest trail (−0.413 ± 0.103, *t* = −4.01). The highest-ranked faecal chlorophyll model, with only the effect of trails, also suggested a strong negative effect of distance from trails on grazing (−0.412 ± 0.103, *t* = −3.99) with similarly sized effects of Julian day, scat water and scat age but no effect of roads (Table 2). The two top models accounted for 0.97 of the Akaike weight, with all remaining models accounting for no more than 0.03 of the Akaike weight. Interestingly, the chlorophyll model with NDVI, an index of the greenness of the landscape strongly sensitive to plant chlorophyll abundance, was poorly ranked overall (Table 2).

## Discussion

We found strong associations between faecal cortisol and faecal chlorophyll and between faecal chlorophyll and spatial attributes, particularly the location of roads and trails. This may suggest an important role for spatial heterogeneity in the abundance of nutrient dense but sparse foods (animals, seeds, fruit) as well as grasses and forbs, which are less nutritious (Pritchard and Robbins, 1990) but more commonly consumed by Yellowstone bears (Fortin *et al.*, 2013; Gunther *et al.*, 2014). Additionally, we could not rule out an explicit effect of anthropogenic activity due to the positive collective associations between trails and faecal chlorophyll and between faecal chlorophyll and faecal cortisol. The top model suggested grazing generally increased with proximity to trails. We suspect this may partly reflect the general distribution of trails along valley bottoms, lake shores and stream edges where graminoids and forbs may also be more dense (Wallace *et al.*, 1995) and the associated stress response could be nutritionally mediated. Others have noted that female bears may increase their spatio-temporal overlap with humans, which male bears tend to avoid (Nevin and Gilbert, 2005; Steyaert *et al.*, 2016), perhaps because male bears pose a risk to cub survival (Ben-David *et al.*, 2004) that would suggest trail proximity itself could be an external stressor. However, our models of faecal cortisol that considered distance to roads and trails performed poorly (Table 1). Complex spatio-temporal variation in the distribution of humans and the abundance of other food sources in Yellowstone limits the inferences we can draw from our results as to why grazing is correlated with a stress response. For example, most whitebark pine stands in this ecosystem tend to be far from roads and trails (Mattson *et al.*, 1992; Haroldson and Gunther, 2013; Costello *et al.*, 2014) and bear occurrences near roads appears to increase in years with poor whitebark pine seed production (Haroldson and Gunther, 2013). Disentangling the interaction between food abundance, grazing behaviour and stress response will require additional study.

**Table 2:** Model selection results for generalized linear mixed-effects model of faecal chlorophyll in black bear and grizzly bear faecal samples collected opportunistically from road and trail-side bears and from site searches at GPS collar locations in Yellowstone National Park in 2007–2009

Faecal chlorophyll model description^a^	Model category^b^	k	AICc	ΔAICc	Weight	Log likelihood
day + day^2^ + scat age + scat water + trail	S	8	1283.76	0.00	0.56	−633.67
day + day^2^ + scat age + scat water + trail + road	S	9	1284.41	0.65	0.41	−632.94
day + day^2^ + scat age + scat water + age + sex + species + elevation + NDVI + density + trail + road + cover	G	17	1290.86	7.10	0.02	−627.51
day + day^2^ + scat age + scat water + cover	S	9	1293.78	10.02	0.00	−637.63
day + day^2^ + scat age + scat water + density	S	8	1294.25	10.49	0.00	−638.91
day + day^2^ + scat age + scat water + elevation + NDVI + density + cover	S	12	1294.64	10.88	0.00	−634.86
day + day^2^ + scat age + scat water	N	7	1297.08	13.32	0.00	−641.38
day + day^2^ + scat water	N	6	1297.59	13.83	0.00	−642.67
day + day^2^ + scat age + scat water + road	S	8	1297.91	14.15	0.00	−640.75
day + day^2^ + scat age + scat water + age	B	8	1298.46	14.69	0.00	−641.02
day + day^2^ + scat age + scat water + sex	B	8	1298.91	15.15	0.00	−641.25
day + day^2^ + scat age + scat water + species	B	8	1299.11	15.35	0.00	−641.35
day + day^2^ + scat age + scat water + NDVI	S	8	1299.17	15.41	0.00	−641.38
day + day^2^ + scat age + scat water + elevation	S	8	1299.18	15.41	0.00	−641.38
day + day^2^ + scat age + scat water + age + sex + species	B	10	1302.39	18.63	0.00	−640.87
day + day^2^	N	5	1316.24	32.48	0.00	−653.03
day + day^2^ + scat age	N	6	1317.59	33.83	0.00	−652.67
Intercept only	N	3	1368.84	85.07	0.00	−681.38

^a^Model components are Julian day (day), age class of bear (age), sex of bear (sex), species of bear (species), NDVI, distance to the nearest road (road), distance to the nearest trail (trail), cover (grassland, shrubland and forest), the weathering time between sample collection and defecation (scat age), water content of sample (scat water), the elevation of the sample collection (elevation) and an index of the density of grizzly bears (density).

^b^Candidate models were ordered into the following four categories: global models (G), null models (N), spatial models (S) and bear models (B).

Diet composition is known to affect faecal hormone excretion rates due to factors associated with faecal chemistry and digesta kinetics that are unrelated to endocrine function (Wasser *et al.*, 1993). We do not think the positive association between faecal cortisol and faecal chlorophyll was such an artefact because (i) high-fibre diets are retained for much shorter lengths of time (5–7 hours) than low-fibre diets (e.g. meat: 13 hours) in the gut of grizzly bears and black bears (Pritchard and Robbins, 1990; Elfström *et al.*, 2013) minimizing scat exposure time to circulating hormones in the blood; (ii) high-fibre vegetative diets actually lower faecal excretion rates of steroid hormones in other mammals (Wasser *et al.*, 1993); and (iii) we found identical effect sizes of faecal chlorophyll on cortisol across both bear species and all scat types (Fig. 4). Our results from faecal samples suggest that grazing may be a nutritionally mediated stressor or is associated with an external stressor in Yellowstone bears that operates over a relatively fine temporal scale.

**Figure 5:**
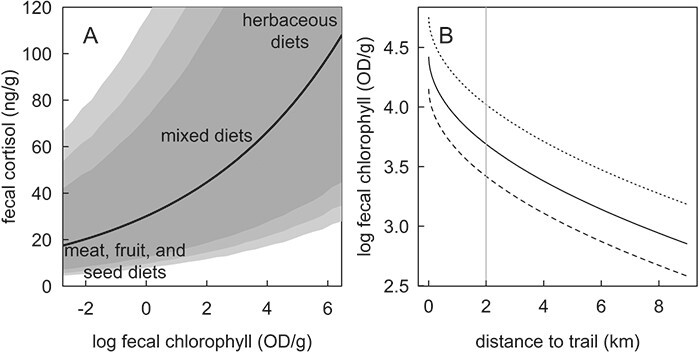
The expected faecal cortisol (with 95%, 90% and 80% CI) in black bears and grizzly bears in Yellowstone National Park over the range of observed faecal chlorophyll levels fit from the top-ranked generalized linear mixed effects model of cortisol with all other covariates fixed at their mean. The potential physiological consequences of substituting high-energy diets for low-energy herbaceous diets (primarily graminoids and forbs) are superimposed on the trendline. Panel B shows expected values of faecal chlorophyll over the range of observed distances to trails (truncated at the 97.5th percentile) fit from the top ranked generalized linear mixed effects models of faecal chlorophyll with all other covariates fixed at their mean values to generate the expected values. The effect of distance to trails at the minimum (0.003 km) and 97.5th percentile (31.9 km) of observed distances to roads are illustrated by the dashed and dotted lines, respectively. The expected faecal chlorophyll with increasing distance to trail at the median (6.4 km) distance to trails of 10 000 random points across all of Yellowstone National Park is also shown (solid line). The median distance from trails (2.0 km) across all of Yellowstone National Park as estimated using 10 000 random points in a GIS is also shown (grey vertical line).

Cortisol levels above baseline circadian and circannual levels or population means are commonly used to infer adaptive responses to an external stressor (Romero, 2004; Ware *et al.*, 2013; Wilson *et al.*, 2020) and, increasingly, potentially maladaptive responses to anthropogenic stressors (Van Meter *et al.*, 2009). Direct comparison of cortisol levels among studies is not possible because of variation in hormone analysis methods and differences in baseline levels between populations. However, we can compare responses and the direction of differences within our population to other studies to broaden inference. Diets high in energy-dense foods have been shown to correspond with lower cortisol levels in other bear populations, suggesting that nutritional state may feedback on bear behaviour or endocrine function (Bryan *et al.*, 2013; Stetz *et al.*, 2013). In coastal British Columbia, lower cortisol was found in the hair of brown bears consuming diets high in salmon when compared to bears that were not feeding on salmon (Bryan *et al.*, 2013) even though salmon streams place bears at risk of antagonistic or even predatory encounters with other bears (Ben-David *et al.*, 2004). In Alaskan brown bears, faecal glucocorticoids were also higher in faecal samples of grass or mixed composition than in samples with meat or berries (von der Ohe *et al.*, 2004). Our results suggest that a significant portion of extra-circannual variation in bear HPA activity is related to variation in diet quality that operates at fine spatio-temporal scale and that other intrinsic (age, sex) and extrinsic (elevation, bear density) factors are less important.

Diets composed mostly of herbaceous vegetation are generally agreed to be less nutritious for bears than diets that include more animal, fruit or seed material that bears must consume to survive or breed in most years (Pritchard and Robbins, 1990; Rode *et al.*, 2001; Robbins *et al.*, 2004; Costello *et al.*, 2016). Cortisol is known to play an important role in normal circadian and circannual rhythms that regulate gluconeogenesis and energy mobilization to meet energy deficits in bears (Harlow *et al.*, 1990; Ware *et al.*, 2013). It seems likely that higher faecal cortisol with grazing indicates an adaptive metabolic response to hypoglycemia in an effort to maintain energy supply to muscles (Sapolsky *et al.*, 2000; Reeder and Kramer, 2005). More severe undernutrition from chronic grazing may also be capable of triggering a more pronounced stress response, as the highest glucocorticoid concentrations are often observed in the most severely undernourished bears (Macbeth, 2013). In our study, the highest cortisol concentrations, by an order of magnitude or more, were found in bear scats composed mostly of vegetation (Fig. 3).

Faecal chlorophyll itself ranged across several orders of magnitude in concentration (Fig. 3). Because the highest observed faecal chlorophyll concentrations were very similar to elk, a sympatric grazer, we posit we sampled from bears whose recent diet was composed entirely of highly photosynthetic leaves from forbs and graminoids. Between the upper and lower bounds of the observed range, (log) faecal chlorophyll may be inversely proportional to the abundance of more nutrient-dense foods in the diet. If bears regularly consume significant quantities of a lower quality, non-photosynthetic food items, such as senescent grasses, then faecal chlorophyl must be more cautiously interpreted (Christianson and Creel, 2009).

There is concern over ongoing shifts in the availability of nutrient-dense food sources for bears including ungulates, cutthroat trout and white-bark pine seeds due to anthropogenic factors (Fortin *et al.*, 2013; Costello *et al.*, 2014; Teisberg *et al.*, 2014). Given their wide dietary breadth and adjustability, bears may be compensating for these shifts through increased herbivory and fall use of ungulates (Fortin *et al.*, 2013; Gunther *et al.*, 2014; Ebinger *et al.*, 2016) as the grizzly bear population growth was stable at the time of this study (van Manen *et al.*, 2013). Our results reinforce the inferences of Stetz *et al*. (2013) that lower quality, herbaceous bear diets are correlated with higher levels of faecal cortisol. However, we were also able to show that a correlation between faecal vegetation and cortisol persisted in bear scats that also included other, more nutrient-dense foods. This result suggests that depressed dietary nutrition might not completely explain the apparent stress response to grazing (Fig. 5). To resolve this question, further research is needed to parse whether endocrine stress responses to grazing reflect physiological consequences of ingesting herbaceous vegetation or a response to intrinsic and extrinsic factors associated with grazing behaviour. Because faecal chlorophyll was such a strong predictor of faecal cortisol, we caution that high faecal, hair or serum cortisol measurements in apparent response to anthropogenic activity or other apparent stressors should be cautiously interpreted if variation in diet composition goes unmeasured. Finally, faecal chlorophyl in bears proved to be an informative, non-invasive metric of foraging behaviour that was relatively easy to measure and interpret as has been found in several herbivores (Christianson and Creel, 2009). The application of faecal chlorophyll as a metric of diet composition and diet quality shows promise for investigating how access to green vegetation may be influenced by climate change, anthropogenic activity or changes in community structure.
